# Bioaugmentation: An Emerging Strategy of Industrial Wastewater Treatment for Reuse and Discharge

**DOI:** 10.3390/ijerph13090846

**Published:** 2016-08-25

**Authors:** Alexis Nzila, Shaikh Abdur Razzak, Jesse Zhu

**Affiliations:** 1 Department of Life Sciences, King Fahd University of Petroleum and Minerals (KFUPM), P.O. Box 468, Dhahran 31261, Saudi Arabia; 2 Department of Chemical Engineering, King Fahd University of Petroleum and Minerals (KFUPM), Dhahran 31261, Saudi Arabia; srazzak@kfupm.edu.sa; 3 Department of Chemical and Biochemical Engineering, University of Western Ontario, London, ON N6A 5B9, Canada; jzhu@uwo.ca

**Keywords:** bioaugmentation, biodegradation, bioremediation, industrial wastewater, pollution, bacteria, quorum sensing, nanotechnology, protozoan grazing, bacteriophage, cell-immobilization, transfection and plasmid transfer

## Abstract

A promising long-term and sustainable solution to the growing scarcity of water worldwide is to recycle and reuse wastewater. In wastewater treatment plants, the biodegradation of contaminants or pollutants by harnessing microorganisms present in activated sludge is one of the most important strategies to remove organic contaminants from wastewater. However, this approach has limitations because many pollutants are not efficiently eliminated. To counterbalance the limitations, bioaugmentation has been developed and consists of adding specific and efficient pollutant-biodegrading microorganisms into a microbial community in an effort to enhance the ability of this microbial community to biodegrade contaminants. This approach has been tested for wastewater cleaning with encouraging results, but failure has also been reported, especially during scale-up. In this review, work on the bioaugmentation in the context of removal of important pollutants from industrial wastewater is summarized, with an emphasis on recalcitrant compounds, and strategies that can be used to improve the efficiency of bioaugmentation are also discussed. This review also initiates a discussion regarding new research areas, such as nanotechnology and quorum sensing, that should be investigated to improve the efficiency of wastewater bioaugmentation.

## 1. Introduction

Industries require a supply of clean water, while at the same time, they generate huge amounts of wastewater that is contaminated with various toxic compounds. In the past, such a situation (high demand of clean water and production of wastewater) only occurred in the developed world, but is now becoming a burgeoning problem in the developing world too, as the result of growing industrialization. For instance, China, one of the fastest growing industrial countries in the world, has generated more than 20 billion m^3^/year of wastewater in the recent years [1]. 

This need to supply a large amount of clean water for industrial activities compounds the challenges that human beings face for providing the same clean water to the ever-increasing human population. Because the supplies of freshwater is limited, especially in countries with a limited rainfall pattern, including North Africa, the Middle East, Southern Europe, Australia, and the Southern and Western states of the USA [2], the reuse of both domestic and industrial wastewater, remains the most feasible long-term solution to this problem [3]. 

**Figure 1:**
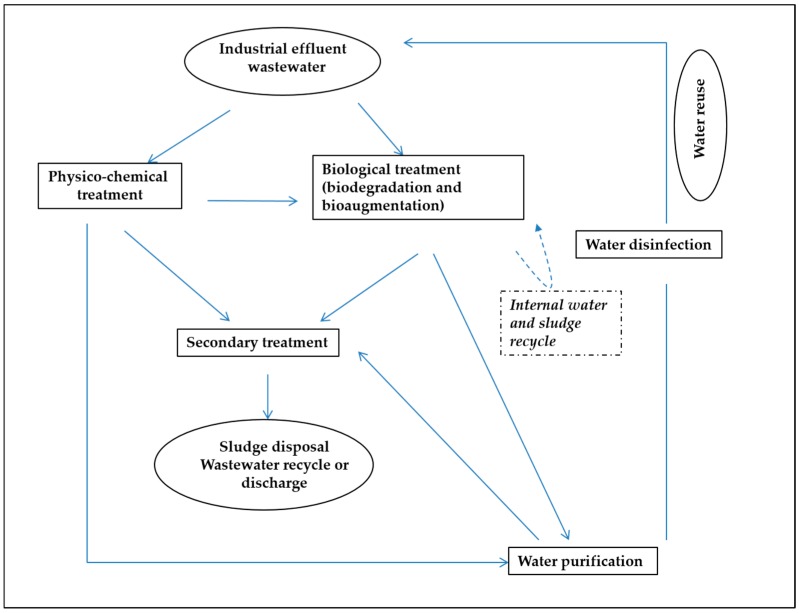
Generic flow of industrial wastewater treatment plan (adapted and modified from [4]).

The contaminated wastewater needs treatment(s) to remove or lower the concentration of pollutants to acceptable levels prior to its reuse or discharge to the environment. With the increase in the awareness of pollutants’ consequences on human health and the environment, all over the world, legislations on the discharge of pollutants are being tightened. As the result, strategies to improve the efficiency of treatment plants to clean industrial wastewater are being developed. Figure 1 summarizes a generic industrial treatment plant. The first steps involve physico-chemical treatment for the removal of organic or inorganic pollutants, and/or biological treatments (removal of organic pollutants), followed by a secondary treatment. This secondary treatment leads to the generation backwash effluents, sludge and membrane concentrates. Backwash effluents can be discharged or sent to a local sewage treatment plant if the discharge criteria are met. Depending upon the type of contaminations, the products of physico-chemical and biological treatments will be subjected to purification and disinfection prior to reuse [4].

In the physico-chemical treatment, approaches including advanced oxidation, nanofiltration, reverse osmosis filtration, and activated carbon filtration are used in removing pollutants; however these processes still remain costly, especially in the context of full scale treatment [5,6,7]. In addition, some of these approaches generate by-products that are toxic to the environment.

Biological treatment is based on the biodegradation of organic pollutants by microorganisms present in wastewater or activated sludge (AS, Figure 1). However, many pollutants, especially highly complex compounds, are not efficiently biodegraded by microorganisms; they may be resistant to biodegradation, and consequently persist in the wastewater, thus compromising water quality. To overcome these limitations, bioaugmentation strategies may be used. Bioaugmentation is the addition of microorganisms that have the ability to biodegrade recalcitrant molecules in the polluted environment. This approach is less-costly and friendlier to environment compared to the physico-chemical approaches. The literature has reported many examples of this approach for the removal of contaminants in soil, and we refer the readers to the following excellent reviews on this topic [8,9,10,11,12,13,14]. Bioaugmentation approaches have been reviewed recently, with an emphasis on operational challenges and wastewater plant management [15]. The current review focuses on the use of bioaugmentation on industrial wastewater exclusively, with an emphasis on microbiological aspects of bioaugmentation, and the biodegradation of recalcitrant organic pollutants found in industrial wastewater. We also intend to identify knowledge gaps for future research efforts. The pollutants discussed are chlorinated molecules, quinolines, dyes, polyaromatic compounds, gycol-ether, cyanide and nitrogen heterocyclic compounds. The pollutants commonly found in domestic wastewater such as carbohydrates, lipid and proteins, and nitrate are excluded from this review. In addition, limitations of bioaugmentation strategies are presented, and key parameters that affect biodegradation efficiency and potential new areas, such as nanotechnology and quorum sensing, which could be exploited to improve the success of industrial wastewater bioaugmentation are also discussed.

## 2. Bioaugmentation to Remove Recalcitrant Pollutants in Industrial Wastewater

Wastewater activated sludge contains naturally occurring microorganisms that biodegrade a wide range of pollutants, but as previously mentioned, some pollutants are resistant to biodegradation. Several factors account for this resistance: high toxicity, low water solubility, low bioavailability, high stability and low biodegradability. Some compounds may not be efficiently used as substrates by microbial metabolic enzymes. The chemical structures of certain pollutants may be so complex that consortia of different microorganisms may be necessary for their biodegradation, or all of the microorganisms necessary may not be simultaneously present in the environment. In many cases, recalcitrant compounds may be new, and as a result, microorganisms may not have yet adapted to use them as a substrate [16]. Bioaugmentation can overcome these challenges, as one of its main advantages is that treatment can be tailored to a specific pollutant that is dominant in the environment. Thus, this approach is attractive for addressing both the increasing number of emerging pollutants as well as pollutants that are present at high concentrations. Over the last decade, many investigations have been dedicated to testing bioaugmentation strategies to clean wastewater, and most have focused on recalcitrant molecules. Below, examples of the use of bioaugmentation for the removal of pollutants from industrial wastewater from the early 2000s to the present are presented (see also Table 1).

## 3. Applications of Bioaugmentation

### 3.1. Chlorinated and Fluorinated Compounds Removal

Halogenated compounds are used in various applications, such as plastic components, lubricants, adhesives, solvents, degreasing agents, pesticides, fungicides, and wood preservatives [17]. For instance, in 2012, it was estimated that worldwide, the total amount of chlorinated solvents used was 764,000 metric tons [18]. Such extensive use in both industry and homes leads to contamination of wastewater, and bioaugmentation has proven to be an important strategy for their elimination. The bacteria *Acinetobacter* sp. TW and *Comamonas testosteroni* I2 were shown to biodegrade 4-fluoroaniline and 3-chloroaniline in synthetic wastewater medium supplemented with AS, respectively [19,20]. In addition, the authors identified optimum conditions that favored colonization and thus biofilm formation that significantly increased biodegradation [20]. The biodegradation of 2,4-dichlorophenol by bioaugmentation with a consortium of bacteria has been reported in a laboratory-scale set-up by using synthetic wastewater enriched with AS [21]. Recently, using a fluidized bed biofilm reactor (FBBR) and expanded granular sludge bed (EGSB), an increase in the biodegradation of 2,4,6-trichlorophenol following bioaugmentation with *Desulfitobacterium* sp. has been reported [22]. However, it is interesting to note that these aforementioned studies were carried out at laboratory scale only. Therefore, the removal of chlorinated molecules by bioaugmentation still remains to be evaluated in the context of full scale wastewater treatment plant.

### 3.2. Lignin Removal

Another successful study of bioaugmentation was carried out in wastewater treatment for the paper industry. The pulp and paper industry generates large volumes of wastewater with a high lignin content, known as black liquor. For instance, it is estimated that seven tons of black liquor are produced per ton of pulp generated [23]. Black liquor is a mixture of complex compounds, including lignin, polysaccharides and resinous compounds. Natural biological treatment with AS cannot efficiently remove these compounds because lignin-biodegrading microorganisms are not commonly found in wastewater [24]. Thus, selection and addition of lignin-biodegrading microorganisms into wastewater provides an attractive strategy to remove specific pollutants originated from black liquor. Zheng et al. [25] tested a consortium of lignocellulose-biodegrading microorganisms isolated from AS in a sequencing batch reactor (SBR). This mix of microorganisms, which were reported elsewhere [26], were *Comamonas* B-9 and *Pandoraea* B-6 (bacteria), and *Aspergillus* F-1 (fungus). The results showed that the bioaugmented AS significantly enhanced the removal of lignin (>50%) in a laboratory set-up consisting of a SBR, with a maximum working volume of 2 L. All these investigations show that bioaugmentation is a feasible alternative strategy to enhance the biological treatment of wastewater with a high lignin content [25]. However, the scaling up of this process in the context of wastewater treatment plant awaits evaluation.

### 3.3. Quinoline and Pyridine

Quinolines and pyridines are *N*-heterocyclic aromatic compounds commonly found in industrial and pharmaceutical raw materials and used as solvents for dyes, paints, and wood treatment chemicals, which leads to their presence in industrial wastewater. Quinolines are also present in coal tar and petroleum products. They persist in the environment because of their low biodegradability, and they are carcinogenic. A report showed the enhancement of quinoline biodegradation by using *Bacillus* sp. isolated from soil in a 250 mL batch reactor, filled with petroleum refinery wastewater [27]. A study reported the biodegradation of quinoline in wastewater bioaugmented with *Burkholderia pickettii* [28], and another one evaluated, with success, the biodegradation of quinoline and pyridine using wastewater medium bioaugmented with *Paracoccus* sp. and *Pseudomonas* sp. [29]. In the later study, although the concentrations of quinoline and pyridine were reduced, however, the nitrogen content remained high. To address this limitation, the same mixed biodegrading bacteria were tested in a 250 mL SBR reactor containing a modified zeolite. Zeolites contribute to the removal of nitrogen content by adsorption. The results showed a reduction of quinoline and pyridine concentrations along with nitrogen content in the medium [30]. The removal of the two *N*-heterocyclic compounds pyridine and quinoline after bioaugmentation of 4 bacterial strains (*Paracoccus* sp. BW001, *Shinella zoogloeoids*, *Pseudomonas* sp. BW 001 and *Pseudomonas* sp. BC 003) was also evaluated in coking wastewater [31]. The same research group also reported the ability of the mixed bacteria *Paracoccus* sp. and *Pseudomonas* sp. to remove pyridine, quinoline and ammonium in a laboratory scale bioreactor consisting of a zeolite-biological aerated filter [32]. Recent investigations have shown an increase in pyridine removal following the bioaugmentation of industrial wastewater with *Rhizobium* sp. using a SBR [33] and *Paracoccus denitrificans* in a membrane batch reactor [34]. Up to date, no report has been made on the application of this approach in field conditions for the removal of pyridine and quinoline.

### 3.4. Synthetic Dyes

Synthetic dyes, which primarily consist of azo- and anthraquinone-based molecules, are extensively used in textile and cosmetics, and over 7 × 10^5^ tons of dyes are produced per year. It is estimated that 2%–10% contaminate the environment, primarily through industrial wastewater [35]. Azo-dyes, which are the largest and most diverse group of dyes, are generally resistant to biodegradation with conventional AS treatment [36]. The removal of an azo-dye, Acid Orange 7, by bioaugmentation with *Shewanella* sp. XB, was evaluated in a 2 L membrane-aerated biofilm reactor, with encouraging results [37]. The synthesis of anthraquinone-dyes requires bromoamine acid (BAA), as the major synthetic intermediate [38]. The industrial production of BAA, to meet the supply of anthraquinone-dyes, leads to the generation of wastewater contaminated with BBA, and around 20 m^3^ of wastewater are discharged per ton of BAA produced [38]. This compound is toxic and resistant to biodegradation; BAA-biodegrading *Sphingomonas* sp. strain was isolated and bioaugmented in a laboratory combined process of microelectrolysis and biological aerated filtration of contaminated wastewater [39]. Another strain of the *Sphingomonas* genus, *Sphingomonas xenophaga*, was isolated and used successfully at laboratory scale for the removal of BAA in bioaugmentation studies with synthetic wastewater medium [40,41,42]. However, so far, studies are still needed to establish whether these encouraging results on the removal of synthetic dyes could be extended to full scale treatment plant.

### 3.5. Cyanides

Cyanides are one of the most toxic compounds released from coal during the coking process in the steel industry [43]. Thus, this industrial wastewater must be treated before being discharged into the environment. To enhance the efficiency of the biological removal of cyanides, bioaugmentation was applied to a full-scale coke wastewater treatment process by using cyanide-degrading yeast *Cryptococcus humicolus* and unidentified cyanide-degrading microorganisms in wastewater that contained ferric cyanide. However, this process was of limited efficiency as a result of poor settling performance of microbial flocs and the slow biodegradation rate of ferric cyanide in wastewater [43]. This is one of first reports on the evaluation of bioaugmentation in full scale treatment plant, and clearly, more investigations are needed to make this approach efficient in the context of cyanide removal. 

### 3.6. Nicotine

The tobacco industry is associated with the release of a substantial amount of wastewater containing various toxic substances [44], one of which is nicotine, a possible carcinogen [45]. For every ton of cigarettes produced, 60 tons of contaminated wastewater are discharged [46]. For instance, more than five trillion cigarettes were produced worldwide in 2009 [47], and with a weight of 1 g/cigarette, the total amount of wastewater produced was more than 300 million tons in 2009. Bioaugmentation has been evaluated as a strategy to remove these pollutants. Studies have identified several bacteria capable of degrading nicotine, including *Acinetobacter* sp. and *Sphingomonas* sp. [48]. By using a 2-L synthetic wastewater reactor that contained COD (3200 mg/L), nicotine (1 g/L), and AS from a wastewater treatment plant, Wang et al. [49] tested the effect of bioaugmentation with *Acinetobacter* sp. on the biodegradation of nicotine. The results showed a significant increase in nicotine removal from ~10% in the control reactor to 98% in the bioaugmented reactor. Interestingly, this removal of nicotine was associated with a significant increase in total bacteria and a decrease in COD in the bioaugmented reactor [49]. Nicotine is toxic to bacteria, and therefore, its removal also promotes bacterial growth, which in turn augments the overall biodegradation process. Similar results were reported with another nicotine biodegrading strain, *Pseudomonas* sp. HF-1, in a sequencing batch reactor used to treat tobacco wastewater [50]. These studies illustrate the benefit of bioaugmentation in eliminating nicotine. However, the aforementioned investigations were carried out in small scale conditions only, and up to date, there is no report on the use of this approach in the context of tobacco wastewater treatment plant. 

### 3.7. Diethylene Glycol Monobutyl Ether (DGBE)

Glycol ethers, mainly ethylene glycol monobutyl ether and diethylene glycol monobutyl ether (DGBE) are polar solvents that are miscible with both organic chemicals and water, and are commonly used in paints and cleaners. These compounds are toxic in animal models [51], and are refractory to biodegradation, thus accumulate in the environment after their discharge in industrial wastewater [51]. Recently, Chen et al. [51] evaluated the potential of a strain of *Serratia* sp. to remove DGBE in the context of bioaugmentation of contaminated wastewater from a silicon plate industry. The results have shown the increase in DGBE removal at both laboratory- and full-scale [52].

### 3.8. Polycyclic Aromatic Hydrocarbons and Heterocyclic Compounds

Another group of important pollutants frequently found in industrial wastewater are polycyclic aromatic hydrocarbons (PAHs). They are primarily found in petroleum products, but also in many waste streams from various industrial processes, such as coal conversion and synthesis of organic chemicals. These polycyclic aromatic molecules are recalcitrant to biodegradation, thus, they persist longer in the environment, with the attendant consequences on toxicity to animal and the environment. One of the PHAs is naphthalene. Its removal has been tested in the context of bioaugmentation in coal gasification wastewater, with the use of a strain of *Streptomyces* sp., in a membrane bioreactor, which showed a significant removal of naphthalene [53]. A similar study was carried out on the bioaugmentation of coking wastewater with a consortium of *Paracoccus denitrificans* and five of *Pseudomonas* sp. strains. The bioaugmentation facilitated removal of naphthalene, phenol, pyridine, quinoline, and carbazole present in the coking wastewater [54]. 

Another bioaugmentation experiment has been reported for the removal of phenols, naphthalenes, carbazole, dibenzofuran and dibenzothiophene, which are all products found in real coking wastewater. In this investigation, zeolite-biological aerated filters (Z-BAFs), with *Arthrobacter* sp. (free and immobilized) were employed, and the results showed a significant increase in pollutant removal in bioaugmented batch reactors, and higher removal rate was reported with immobilized bacteria [55]. A study reported the ability of a mixture of phenol-degrading bacteria in removing phenol present in coal gasification wastewater using biological contact oxidation reactor [56]. However in this study, information on the species of bacteria was not provided [56]. 

## 4. Limitations of Bioaugmentation Technologies

This review shows that the concept of bioaugmentation in wastewater has been extensively investigated at the laboratory scale with encouraging results. However, this success has not been translated to full scale wastewater treatment. In general, the removal of pollutants by bioaugmentation has been investigated in soil, surface water and groundwater. While the usefulness of bioaugmentation has been reported, a sizable number of failures of bioaugmentation have also been documented [57,58]. One successful full scale bioaugmentation story that has been reported is the in in-situ removal of chlorinated solvents (primarily chlorinated ethenes) in groundwater, with the use of anaerobic bacteria of *Dehalococcoides* group. Readers are referred to an excellent book on this topic [59].

Studies often observe that the number of exogenous microorganisms decreases shortly after addition to a site. There are several explanations for the death of introduced microorganisms, including both abiotic and biotic stresses. The stresses happen due to insufficient substrates, temperature changes, pH, nutrient limitations, competition between introduced and indigenous microorganisms, phase infections, shock of pollutant load, grazing by protozoa, and factors associated with quorum sensing (QS), which have all been proposed as possible causes of failure [59,60]. Documented evidence on bioaugmentation failures and strategies that can be used to overcome these limitations in the context of wastewater treatment are summarized in Table 2 and discussed below.

### 4.1. Protozoan Grazing

A study reported the ecological causes of bioaugmentation failure [60]. In a laboratory-scale (2 L) sequencing batch reactor, *Microvirgula aerodenitricans* was added to a synthetic medium that contained acetate and AS (from piggery wastewater) to remove N_2_. However, no difference in N_2_ removal was observed between the bioaugmented and non-bioaugmented reactors. Interestingly, further analyses demonstrated that the added bacteria disappeared from the reactor within 2 days [60] as a result of the growth of protozoa, which destroyed the bacteria; a phenomenon known as protozoan grazing. Failure of bioaugmentation as the result of protozoan grazing has also been reported in the removal of the recalcitrant pollutant 2,4-dichlorophenol from lake waters by *Pseudomonas* sp. [61]. Protozoan grazing has also been shown in AS studies with the use of engineered *Pseudomonas cepacia* transfected with a green fluorescent protein gene [62]. Thus, before bioaugmentation is carried out under real-life conditions, studies to establish whether bioaugmented microorganisms will be able to grow efficiently in the tested environment are required. 

### 4.2. Inoculum Size

The inoculum size is another important factor for successful bioaugmentation. The ability of *Pseudomonas cepacia* to biodegrade p-nitrophenol as a function of bacterial concentration in lake waters was evaluated [63]. The results showed that the bioaugmented bacterium at concentration of <400 cells/mL was unable to biodegrade this pollutant, whereas encouraging results were obtained when bacterial concentrations were in the range of 10^4^–10^5^ cells/mL. Interestingly, further analysis indicated that the failure of the growth of this “low-density bacterial” inoculum (<400 cells/mL) was associated with protozoan grazing, which indicated that the lower the number of seeding bacteria, the higher the possibility of protozoan grazing. Thus, inoculum size is critical to the success of bioaugmentation. For instance in the bioaugmentation of groundwater, an inoculum of 10^6^–10^7^ cells/mL is recommended [57], which could also be adapted in the context of wastewater treatment.

### 4.3. Bacteriophage Infection

Bacteriophages are viruses that infect and destroy bacteria, and they are considered to be the most abundant and diverse biological entities on the earth, with ten phages for every bacterial cell in most studied ecosystems, including wastewater [64,65]. There is evidence showing that bioaugmentation failure can result from infection of bacteria by bacteriophages. For instance, a study reported the effect of phage infection on the nitrifying bacterium *Lutimonas* sp. for the removal of ammonia in wastewater [66]. 

Failure of bioaugmentation was a result of the disappearance of the bacterial strains, which was associated with an increase in the population of phages in the environment [66]. Similar results were obtained regarding the removal of phosphate by *Candidatus accumulibacter* and *Microlunatus phosphovorus* bioaugmented in a wastewater plant [67,68]. Thus, phage infections can have a significant effect on the growth of bioaugmented bacteria. To the best of our knowledge, no sustainable strategies exist to remove bacteriophages from wastewater. However, as discussed earlier, before initiating a bioaugmentation process, the ability of bacteria to grow in the new environment should be established, and various methods can be used to monitor microorganism growth, including plating, the most probable number (MNP), polymerase chain reaction (PCR), quantitative PCR (qPCR) and microarrays, among others [15,57]. For instance in groundwater treatment, during monitoring, if bacterial concentration falls below 10^6^ cells/mL, new inocula need to be added so as to maintain the efficiency of bioaugmentation [57]. Thus, this monitoring should be recommended as a standard practice in wastewater bioaugmentation, and a minimum 10^6^ cell/mL should be maintained throughout the process, as it is the case in groundwater treatment [57].

## 5. Potential Strategy to Improve the Efficiency of Bioaugmentation

### 5.1. Immobilized or Entrapped Cells in Bioaugmentation

To overcome some of the aforementioned limitations, immobilization (entrapment or encapsulation) of microorganisms can be used. This process consists of entrapping living microorganisms within a semi-permeable gel or carrier materials, leading to several advantages over the free cell bioaugmentation: it can protect against protozoa grazing, bacteriophage infections; enhance biological and physical stabilities, by reducing challenges such as sudden and brief variations of temperature or pH; protect from abiotic stresses such as the inhibitory effect of toxic compounds or heavy metals as well as the increase of shear stress. Overall, encapsulation is associated with high biomass concentration and enhanced cell survival. 

This approach has been investigated in the context of bioaugmentation, with successful results in wastewater, when compared to free cell systems (non-immobilized). For instance, the use of immobilized naphthalene-cultivated *Arthrobacter* sp. improved the removal of carbazole, dibenzofuran and dibenzothiophene from coking wastewater [69]. A study reported an increase in removal of nitrogen and phosphate from wastewater by encapsulated *Chlorella* sp. [70], and another one evaluated the removal of azo dyes from synthetic saline wastewater using the immobilized halotolerant bacterium *Bacillus firmus* [71]. Similar results have been reported elsewhere [72,73,74,75]. Though the use of this strategy is associated with increase in bioaugmentation results, however, the process remains costly, and especially when huge volumes of wastewaters have to be treated. 

### 5.2. Quorum Sensing (QS)

Colonization by bioaugmented bacteria is an important determinant in the success of bioaugmentation. For most bacteria, this colonization is ensured by the formation of biofilms, which are structures in which communities of bacteria are attached through a self-produced hydrated polymeric matrix [49]. The formation of biofilms is mediated by a process known as quorum sensing (QS). In QS, bacteria release chemical signals for bacteria-to-bacteria communication, known as auto-inducers, that lead to bacterial cooperation and biofilm formation, and thus to an increase in the bacterial population. The most commonly reported auto-inducers are acylated-l-homoserine lactones (AHLs) [76]. In pathogenic bacteria, this cooperation leads to virulence and therefore to disease development. As a result, a new area of research has opened up based on the inhibition of QS as a strategy to treat bacterial infections, and several QS inhibitors are being investigated [76,77].

The modulation of QS could also contribute to the improvement of bioaugmentation for wastewater treatment, although little research has been carried out in this field. In a first experiment of this kind, the addition of AHLs to an AS was associated with a significant increase in phenol biodegradation rate, from around 10 to 250 µmole/L/h after 14 days of incubation [78]. Similarly, an increase in nicotine removal by bioaugmentation with *Acinetobacter* sp. TW in synthetic wastewater was associated with the expression of mainly short chain AHLs [49]. Other studies have shown the existence of QS in wastewater bacteria, through the production of AHL auto-inducers, and these signaling molecules were shown to regulate the dynamics of the microbial population in bioreactors [79,80]. These observations indicate that microbial communities exhibit QS during wastewater treatment and that this phenomenon can increase the formation of biofilms and colonization, and lead to improved biodegradation of pollutants. However, more research is necessary to establish the dynamics of the interactions between bioaugmentation and QS. For example, a study showed that the environmental conditions of QS for ideal colonization are not necessarily the same as those for the optimum biodegradation of 4-fluoroaniline [49]. Consequently, QS may open the path for improved efficiency of bioaugmentation in the treatment of wastewater. 

### 5.3. Genetically Modified Microorganisms and Gene Transfer

The use of genetically modified microorganisms (GMM) is another approach to improve bioaugmentation. The GMM are transfected with genes that encode catabolic enzymes involved in the biodegradation of pollutants, thus increasing microorganism biodegradation efficiency. The early breakthrough of GMM was reported in the seminal work of [81] on genetic manipulation of oil biodegradation *Pseudomanas* bacterium. Since then, other GMMs have been developed. For instance, *Pseudomonas* sp. and *Pseudomonas putida* strains have been genetically engineered with plasmids containing genes coding for catabolic enzymes used in the biodegradation of monoaromatic compounds. The results showed an increase in the biodegradation of three benzoate derivatives (chlorobenzoate, methyl benzoate, and ethyl benzoate) [82,83]. Genetically modified *Escherichia coli* has been tested for biodegradation of atrazine and direct Blue 71 dye in wastewater [84,85]. The same concept has also been used in wastewater for the removal of heavy metals: mercury with *Pseudomonas putida* [86,87], and cadmium, lead and nickel with *E. coli* [88,89].

Although these studies indicate that GMMs have considerable potential to remove pollutants, serious concerns have been raised regarding their long-term environmental effects [57,90]. Indeed, GMMs present a risk of affecting the natural ecological and environmental equilibrium of microorganisms, and many countries in the world are adopting restrictive legislation against their widespread use in the environment. However, in a well-controlled and confined milieu, these GMM hold great potential as a bioaugmentation strategy. 

On the other hand, the use of purified catabolic enzymes to increase biodegradation has also been proposed. For instance, the enzyme laccase has been used to increase the removal of various wastewater pollutants. However this approach still has some limitations that include the high cost of enzyme production for large scale use, the decreased stability of enzyme in wastewaters as the result of change of enzyme conformation, and the reduced enzyme recovery and reusability among others [91,92]. Some of these setbacks can be overcome by the use of enzyme immobilization and insolubilization, and enzyme based membrane bioreactor [92]. 

### 5.4. Plasmid Mediated Bioaugmentation

The exploitation of horizontal gene transfer (HGT) among bacteria is another approach to improve bioaugmentation. In natural environment, bacteria can acquire new catabolic functions by receiving genes encoding catabolic enzymes from closely or distant related bacteria, through mobile elements such as plasmids or transposons. HGT requires the use of donor bacteria containing plasmids of interest, and these donors will be mixed and cultured with recipient bacteria. Once the transfer takes place (via conjugation or transformation), recipient bacteria become trans-conjugants by acquiring new catabolic biochemical processes [93,94].

The first investigations on the removal of pollutants from wastewater using plasmid mediated bioaugmentation have been reported in the late 1980s. For instance, a donor strain *Pseudomona putida* that harbored a 3-chlorobenzoate catabolite plasmid was evaluated in laboratory scale using AS. Although the transfer of conjugative plasmids to indigenous bacteria was observed, however the results did not show an increase in the biodegradation of 3-chlorobenzoate [95]. Since this early work, several investigations have been dedicated in testing this concept in wastewater treatment, and so far, most of this work has been carried out at laboratory scales (and to a lesser extent, at pilot scale). Readers should refer to the following excellent reviews on this topic [96,97]. Overall, contradictory results have been obtained, some experiments proving the efficacy of this method while others not [96,97]. The following have been the proffered main causes of failure: inability of donor bacteria to persist in the environment, inefficiency of plasmid transfer in recipient bacteria, low number of donor and recipient bacteria, and reduced stability of plasmids once in the recipient bacteria [96,97]. These failures need to be analysed in the context of HGT mechanism [94]. Indeed, the success of HGT depends upon the types of donor bacteria (and its plasmids) and the recipient bacteria. Bacteria can harbor “pilus specificity” and “surface exclusion”, both parameters can affect the binding of donor-recipient bacteria. The type of mechanisms of DNA restriction and anti-restriction systems present in recipient bacteria will either destroy or maintain the new plasmids inside recipient cells. Plasmids can also harbor DNA restriction enzymes on their own, which can affect the integrity of recipient bacterial chromosome. Finally, to persist in the recipient cell, some plasmids need to integrate into bacterial chromosome, thus, processes that control the recombination will also affect HGT success [94]. 

In wastewater, the choice of plasmids and donor bacteria is controlled by the operator (thus, more efficient bacteria and plasmids with best genetic makeup can be selected), however recipient bacteria are part of microbial community present in AS. Thus, understanding genetic characteristics of these recipient bacteria in relation with the aforementioned biological events that affect the success of HGT will be central in improving this plasmid-bioaugmentation approach in wastewater. In this context, new tools such as metagenomics, transcriptomics, proteomics and metabolomics can be helpful in achieving this goal [98,99,100,101].

### 5.5. Nanotechnology in the Context of Bioaugmentation

Nanotechnology, by the production of nanoparticles or nanomaterials (NM), is increasingly becoming a technology with applications in almost all sectors of sciences and technology including, pharmaceutics, medicine and food-industry and agriculture [102]. Several types of NMs have been developed and tested under various conditions. These include titanium dioxide, and zinc oxide, silver and gold nanoparticles, and carbon nanotubes among others. These materials have a size of 1–100 nm, thus providing a large surface area, a feature that tremendously increases adsorption properties, and this can be exploited further by attachment of functional groups so as to increase affinities towards target molecules. This provides an excellent strategy in the removal of both inorganic and organic pollutants from the environment, including wastewater, and readers should refer to these recent reviews on this topic [103,104,105,106]. NM inhibit bacteria growth, and this, for instance, has been exploited as a rational for their use as antibiotics [107]. Therefore, in the context of bioaugmentation, NM a priori do not provide any benefit, since they inhibit microbial population in the contaminated environment. 

However, new evidence is emerging that this approach can tremendously improve bioaugmentation. For instance, a report using carbonanotubes (CNTs) has shown that the inhibition of a bacterium strain *Arthrobacter* sp. growth depends on CNT concentration. Concentrations below 25 mg/L did not affect bacteria growth, while value of CTN >100 mg/L were inhibitory [108]. *Arthrobacter* sp. biodegrades the organic pollutant atrazine, and the use of CNT at concentration >25 mg/L, in a 250 mL-batch reactor, was associated with an increase in the biodegradation of atrazine (compared to the control without CNT). This increase in biodegradation rate was associated with stimulation of bacterial growth, and that at ≤25 mg/L of CTN, *Arthrobacter* sp. can fully utilize atrazine that is adsorbed in CNTs. The other positive effects associated with these NM is that CNT can be reversibly oxidized and reduced, thereby conferring capacity to serve as electron carriers in multiple redox reactions, thus increasing the biodegradation reactions rates [108]. Similar results of increase efficiency of NM to biodegrade organic pollutants (dyes) were reported using effluent wastewater from a textile industry [109].

Another limitation of biodegradation or bioaugmentation is the reduced bioavailability of pollutants. To counteract this limitation, bacteria can be functionalized by fixing on their surface “thermal responsive NM”. Owing to their high surface, NM will favor adsorption of pollutants, and an increase in temperature above the “lower critical solution temperature” will result to a slow release of the adsorbed pollutant in the vicinity of biodegrading bacterium, leading to a better biodegradation. This approach has been successfully tested using phenol as a model compound, in a 250 mL-batch reactor [110]. 

As discussed earlier, immobilization and entrapment of microorganisms can be used to improve bioaugmentation efficiency, however, mass transfer limitation of substrates is still the major drawback in the application of this approach. The use of NM, because of the large surface area, could mitigate these limitations. Recently, this has been tested successfully, with the use of magnetic nanoparticle immobilized-*Rhodococcus rhodochrous* strain for the biodegradation of chlorophenol in a 100-mL batch reactor [111]. 

All the aforementioned reports on NM used in bioaugmentation are still in an early stage of investigation. Those studies are based on laboratory scale, however, they highlight the potential of the fast growing nanotechnology in improving bioaugmentation.

## 6. Conclusions

Bioaugmentation is an attractive strategy for the removal of recalcitrant pollutants from wastewater. This approach has proved to be successful in laboratory investigations, but some challenges still exist, especially for scaling up these processes. To date, the successful use of bioaugmentation in real-world conditions has been in the removal of chlorinated compounds by *Dehalococcoides* bacteria from groundwater. In general, one of the main problems associated with bioaugmentation has been the difficulty in maintaining sufficient numbers of biodegrading microorganisms (at least as high as 10^6^–10^7^ cells/mL) in the environment during the bioaugmentation process. Parameters such as initial inoculum density, protozoan grazing and bacteriophage infections have been singled out as the main parameters associated with low bacterial density in the bioaugmented environment. Thus, attention should be paid to these parameters when setting up large-scale water treatment approaches, and monitoring of bacterial density should routinely be carried out. There is now compelling evidence that QS has a strong bearing on microorganism growth, and investigations have shown that QS can be controlled and regulated by the addition of activators or inhibitors. However, this concept has received little attention in relation to biodegradation of organic pollutants. Plasmid-mediated bioaugmentation also offers a potential in improving the biodegradation of pollutants in wastewater, if the genetic characteristics of recipient bacteria can be well defined in relation with HGT success. In the field of nanotechnology, NM are increasingly being used in remediation of contaminated environments, however so far, the use of this approach to increase the efficiency of bioaugmentation has not been explored yet. Therefore, opportunities exist to improve the biodegradation of pollutants in contaminated wastewater. 

## Figures and Tables

**Table 1:** Examples of bioaugmentation of industrial wastewaters for the remediation of important organic compounds.

Pollutant		Set Up	Medium for Bioaugmentation	Bioaugmented Bacteria	Ref.
3-Chloroaniline	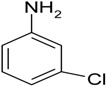	Semi-continuous activated sludge (SCAS) (1 L)	Synthetic influent consisting of skim milk powder	*Comamonas testosteroni*	[19]
4-Fluoroaniline	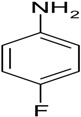	Batch reactor (BR) (250 mL)	Inorganic salt medium	*Acinetobacter* sp.	[20]
2,4-Dichlorophenol (2,4-DCP)	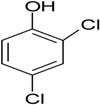	Laboratory-scale continuous flow complete-mixed reactors (CFSTRs) (16 L)	Synthetic wastewater (SW)	Consortium of bacteria	[21]
2,4,6-Trichloro-phenol	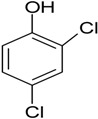	fluidized bed biofilm reactor (FBBR) and expanded granular sludge bed (EGSB)	Industrial wastewater (IW)	*Desulfitobacterium* sp.	[22]
Quinoline	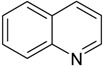	Sequential Batch reactor (SBR) (250 mL)	Petroleum refinery wastewater	*Bacillus* sp.	[27]
Quinoline		SBR (2–7 L)	Coke plant wastewater	*Burkholderia pickettii*	[28]
Pyridine and quinoline	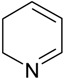	BR (100 mL)	Inorganic medium and wastewater	*Paracoccus* sp. and *Pseudomonas* sp.	[29]
Quinoline and Pyridine		BR (250 mL) with modified zeolite	Coke wastewater	*Paracoccus* sp. and *Pseudomonas* sp.	[30]
Quinoline and Pyridine		SBR	Coke wastewater	Consortium of *Paracoccus* sp. BW001, *Shinella Zoogloeoids* BC026, *Pseudomonas* sp. BC001 and BW003,	[31]
Pyridine		SBR	Industrial wastewater	*Rhizobium* sp.	[33]
Pyridine		2 Membrane Bioreactors (MBR, 25 L each)	Pharmaceutical Wastewater	*Paracoccus denitrificans*	
Acid Orange 7 dye	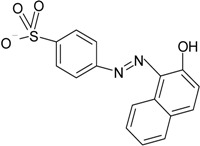	Membrane-aerated biofilm reactor (MABR) (2 L)	SW	*Shewanella* sp. XB	[37]
Bromoamine	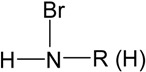	MBR 10 L	SW	*Sphingomonas xenophaga*	[40]
Bromoamine		Combined process of microelectrolysis and biological aerated filter 1–3 L	Wastewater	*Sphingomonas* sp.	[39]
Bromoamine		BR (250 mL)	Inorganic medium	*Sphingomonas xenophaga*	[40]
Cyanide	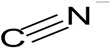	Full scale Cokes wastewater treatment facility (fluidized bed type process) > 3 × 10^5^ L	Cokes wastewater	*Cryptococcus humicolus*, and Unidentified cyanide-degrading microorganisms	[43]
Nicotine	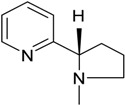	3 reactors of cylindrical shape Height: Bore size of 10:1 (2 L)	Synthetic tobacco wastewater	*Acinetobacter* sp.	[49]
Nicotine		SBR (2 L)	Tobacco wastewater diluted in tap water (7%) (g/mL)	*Pseudomonas* sp.	[50]
Diethylene glycol butyl ether		SBR (2 L) Full scale Plug flow aerated tank (60,000 L)	Wastewater from silicon plate manufacture plant	*Serratia* sp.	[52]
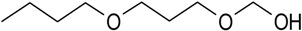					
Lignin (highly complex polymer of phenol)		2 L BR	Industrial wastewater	*Comamonas* and *Pandoraea* (bacteria), and *Aspergillus* (fungus)	[25]
Phenol (PH) and naphthalene (NAP) along with carbazole (CA), dibenzofuran (DBF), and dibenzothiophene	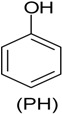	BR (column of 10 × 50 cm)	Coking wastewater from a treatment plant	*Immobilized phenol-utilizing Arthrobacter* sp.	[55]
	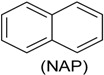				
	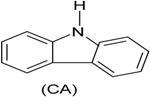				
	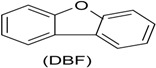				
	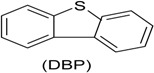				
Naphthalene		MBR (8 L)	Coal gasification wastewater.	*Streptomyces* sp.	[53]
Mixture of phenol, pyridine, quinoline, naphthalene and carbazole		A sequential system of anaerobic reactor(4.9 L), anoxic reactor, A2 (4.5 L), and an oxic MBR (9 L). MBR (9.0 L)	Coking wastewater	Consortium of 6 bacteria containing *Paracoccus Denitrificans* and 5 strains of *Pseudomona*s sp.	[54]
Phenol		Biological contact oxidation reactor (BCOR)	Coal gasification wastewater	Mixture of phenol-degrading bacteria	[56]

**Table 2:** Limitations of bioaugmentation and potential solutions to overcome these limitations for industrial wastewater treatment.

Main Objectives	Limitations	Potential Solutions	Remarks	References
Overcoming low growth or washout of bioaugmented bacteria	Low inocula can lead to limited survival of bioaugmented bacteria	Use of high inocula, at least 10^6^–10^7^ cells per mL. Monitoring of growth of bioaugmented bacteria (followed by the addition of new bacteria)	Has been tested with encouraging results in groundwater	[57]
	Lysis of bacteria by viral (bacteriophage) infections	Monitoring of growth of bioaugmented bacteria (followed by the addition of new bacteria)	Several approaches exist to monitor bacterial levels in wastewater	[15,57]
	Limited bacterial growth as the result of low quorum sensing (QS)	Use of QS inducers to increase bacterial growth. Monitoring of growth of bioaugmented bacteria	Has been evaluated in laboratory scale, but cost may be a limitation in full scale treatment	[78,79,80]
Increase of efficiency of bioaugmentation	Low biochemical ability of bioaugmented bacteria to biodegrade pollutants	Use of genetically modified organisms encoding catabolic efficient enzymes	Has been tested with encouraging results	[84,85,86,87,88,89]
		Use of plasmids encoding catabolic efficient enzymes	Potentially attractive, but so far, not clear evidence of success due to the uncertainty of incorporation of plasmids into receiving organisms	[96,97]
	Low ability of bioaugmented bacteria to biodegrade pollutants	Use of immobilized bioaugmented bacteria	Has been evaluated with encouraging results, but cost may be a limitation in full scale treatment	[72,73,74,75]
		Exploitation of nanotechnology with the use of nanomaterial (NM) along with bioaugmented bacteria to increase biodegradation	NM (at low concentration) increases bacterial growth and the rates of biochemical reactions. Approach is promising but more studies are still needed to ascertain this evidence.	[105,106,108,109]
		Use of functionalized bioaugmented bacteria by fixing NM on their surface to increase bio-availability of pollutants	Promising approach, based one study, thus more studies are needed to support this technology	[110]
